# ECMO for paediatric cardiac arrest caused by bronchial rupture and severe lung injury: a case report about life-threatening rescue at an adult ECMO centre

**DOI:** 10.1186/s13019-022-01856-0

**Published:** 2022-06-06

**Authors:** Xiaoqiong Chu, Weibiao Chen, Yafei Wang, Luqi Zhu, Mengqin Zhang, Sheng Zhang

**Affiliations:** 1 Department of Critical Care Medicine, Taizhou Hospital of Zhejiang Province, Wenzhou Medical University, No. 150, Xi Men Street, Taizhou, 317000 China; 2 Department of Emergency Medicine, Tiantai County Hospital of Chinese Medicine, Taizhou, China

**Keywords:** Bronchial rupture, Children, Cardiac arrest, Veno-venous extracorporeal membrane oxygenation, Case report

## Abstract

**Background:**

Bronchial rupture in children is a rare but dangerous complication after chest trauma and is associated with increased mortality. Veno-venous (V-V) extracorporeal membrane oxygenation (ECMO) is reported as one of the treatments for this life-threatening complication.

**Case presentation:**

A 4-year-old boy with bronchial rupture and traumatic wet lung complicated by cardiac arrest after chest trauma was admitted to an adult ECMO centre. He experienced two cardiac arrests, one before and one during the operation. The total duration of cardiac arrest was 30 min. V-V ECMO was initiated because of severe hypoxia and hypercapnia during the operation. ECMO was performed for 6 days, and mechanical ventilation lasted 11 days. On the 31st day after surgery, he had recovered completely and was discharged without neurological deficit.

**Conclusion:**

V-V ECMO can be considered for supportive care in children with severe acute respiratory failure after bronchial rupture. In an emergency, V-V ECMO can be carried out effectively in a qualified and experienced adult ECMO centre. However, the application of ECMO in children is different from that in adults and requires more refined management.

## Introduction

Bronchial rupture is a rare and life-threatening injury, especially in children, that can lead to varying degrees of pneumothorax, atelectasis, and even death in severe cases. Early treatment has an important impact on the prognosis of traumatic bronchial rupture in children. V-V ECMO can improve oxygenation and ventilation and may play a role as an additional treatment for severe but reversible acute respiratory failure. We present the case of a 4-year-old boy with cardiac arrest caused by bronchial rupture after chest trauma who was successfully rescued through V-V ECMO.

## Case presentation

**Fig. 1:**
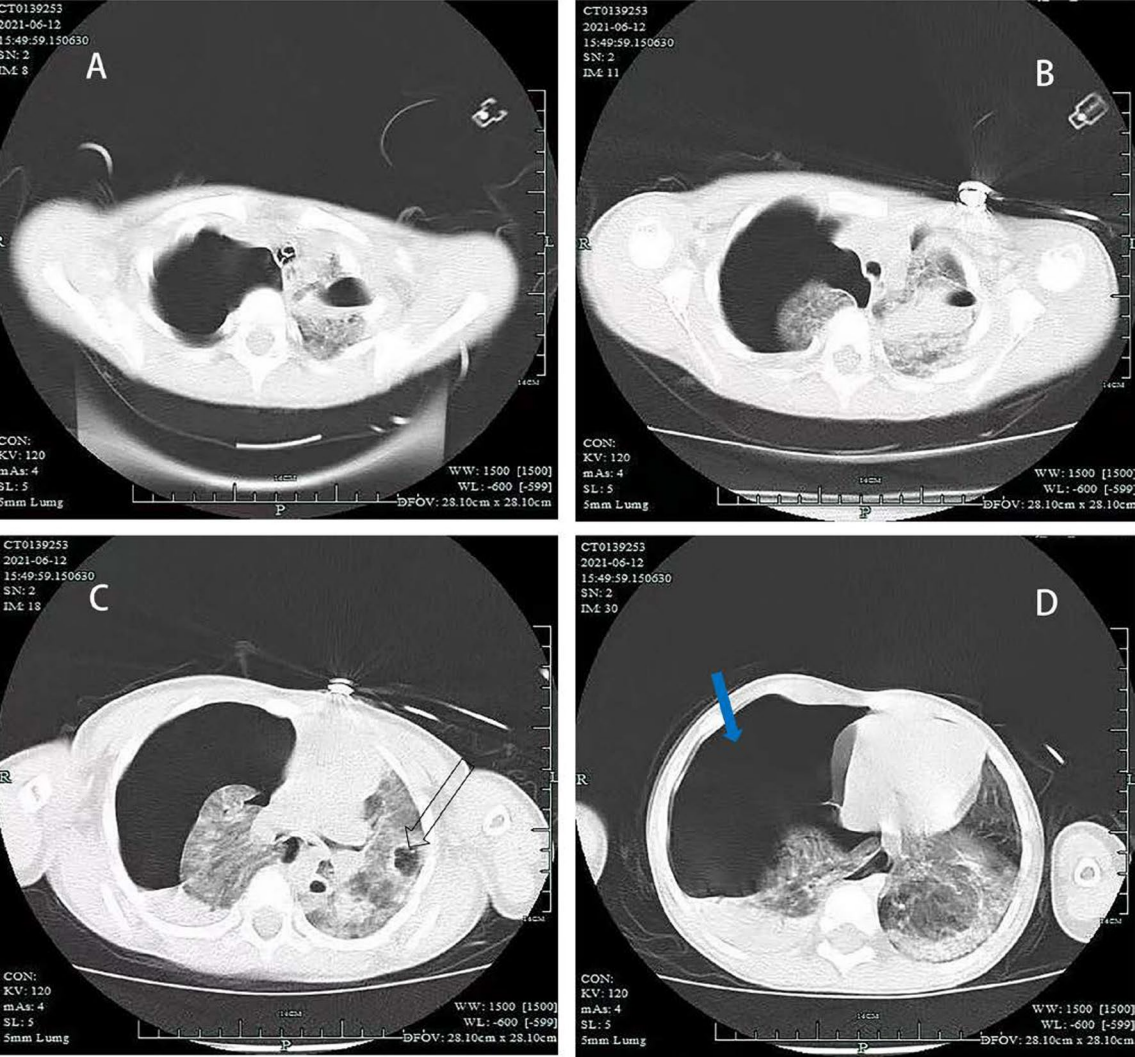
Chest CT (6.12) showed right pneumothorax and right lung compression 90% (blue arrow) and left traumatic wet lung (white arrow)

**Fig. 2:**
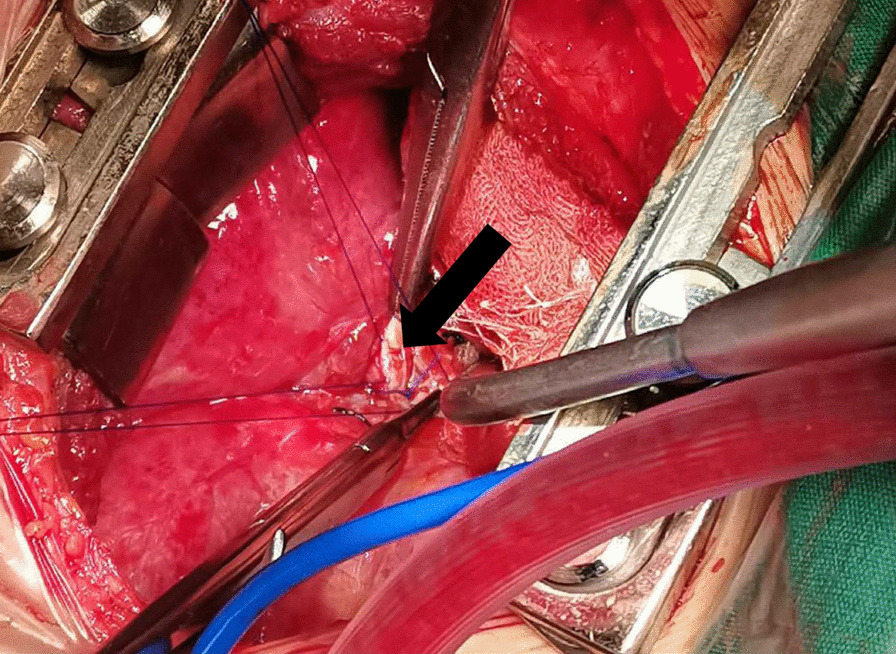
Intraoperative findings. The root of the right middle lobe bronchus was disconnected (black arrow)

**Fig. 3:**
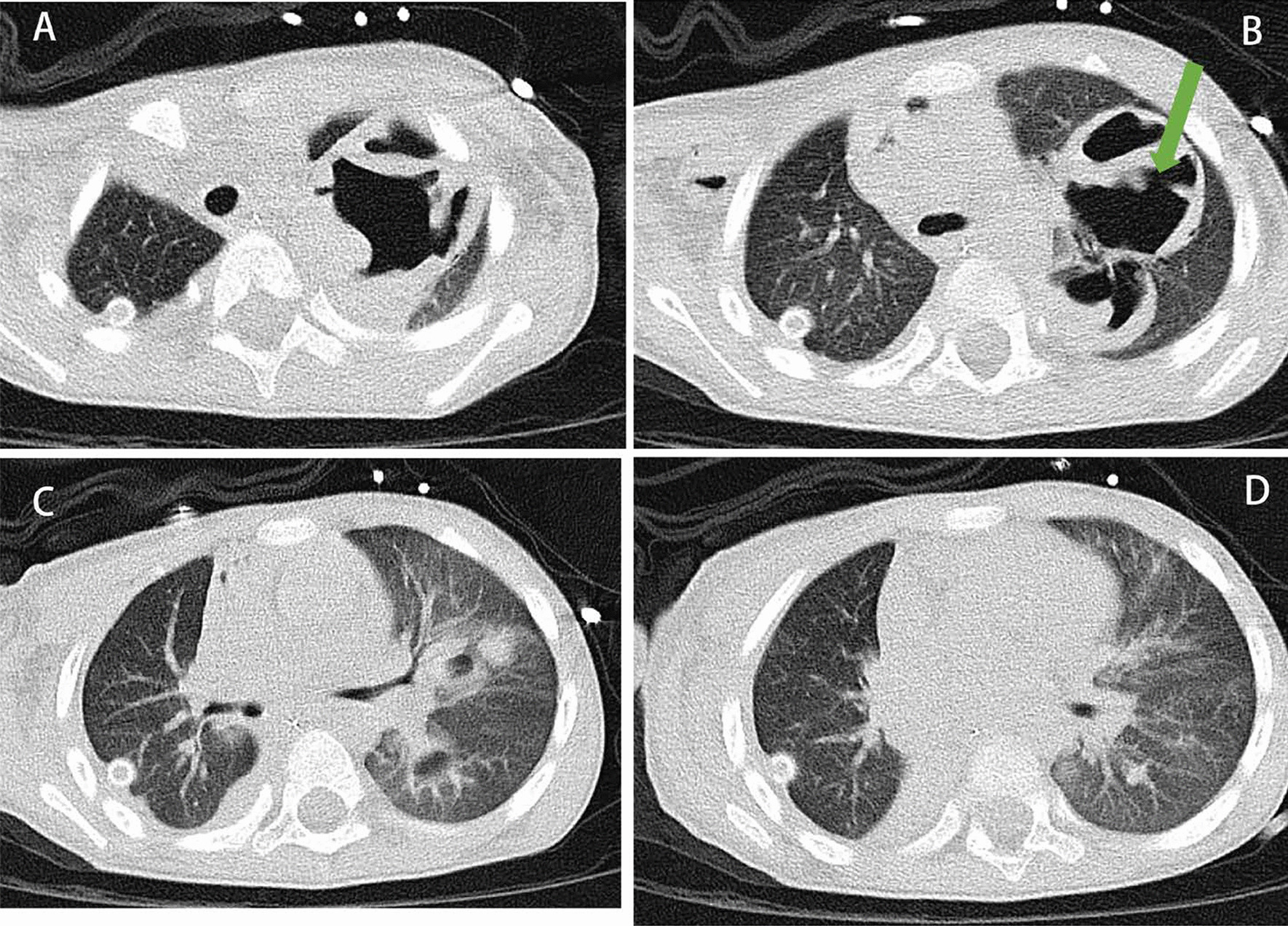
Chest CT (6.24) showed a mass of high-density shadow in the upper lobe of the left lung with cavitation (green arrow)

**Fig. 4:**
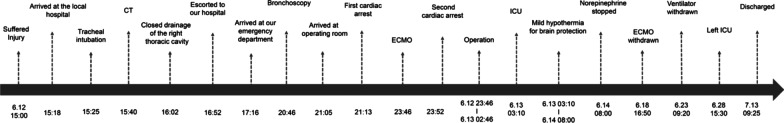
The timeline of the entire treatment process

A 4-year-old boy (height, 100 cm; weight, 16 kg) was hit in the chest by a car. He was conscious and complained of chest tightness. Upon arrival to the local hospital at 18 min after the injury, he was semiconscious and cyanotic. Breath sounds were not heard in the right hemithorax, and his vital signs were unstable: heart rate( HR) of 162 beats per minute (bpm), blood pressure (Bp) of 102/56 mmHg, respiratory rate (RR) of 32 times per minute, and oxygen saturation (S_P_O_2_) of 70%. For sedation, 30 mg of propofol was administered intravenously, and emergency endotracheal tube above the carina was performed. Chest computed tomography (CT) showed right pneumothorax with lung compression of 90%, and the left clavicle was fractured (Fig. 1). A chest tube was positioned in the right thoracic cavity, and a breathing balloon was used for ventilation. His vital signs were as follows: HR of 155 bpm, Bp of 97/50 mmHg, RR of 22 times per minute, and S_P_O_2_ of 98%. Twenty-four minutes later, he was admitted to the Emergency Department of our hospital for further treatment. The results of blood gas analysis were as follows: pH, 7.12; carbon dioxide partial pressure (P_a_CO_2_), 76 mmHg; oxygen partial pressure (P_a_O_2_), 68 mmHg. Bronchoscopy indicated that the right middle lobe bronchus was ruptured. Transthoracic echocardiography ruled out associated blunt cardiac injury. Emergency exploratory thoracotomy and right middle or right middle and lower lobectomy were planned. The patient went into sudden cardiac arrest after being sent to the operating room (S_P_O_2,_ 76%; end-tidal carbon dioxide partial pressure (P_et_CO_2_), 46 mmHg). Return of spontaneous circulation after twelve minutes of external chest compression. He did not regain consciousness. Mainstem intubation of the left bronchus was performed under direct fibreoptic guidance to ventilate the left lung. With pressure control ventilation, the fraction of inspiration O_2_ was 100%, peak pressure was 32 cmH_2_O, and tidal volume was 45 mL; HR was 145 bpm, Bp was 92/48 mmHg (0.05 µg/kg/min norepinephrine), RR was 20 times per minute, and S_P_O_2_ was 70%. Blood gas analysis results at this time were as follows: pH, 6.87; PaCO_2_, 114 mmHg; PaO_2_, 46 mmHg; plasma lactic acid (Lac), 6.7 mmol/L; K + , 3.2 mmol/L; haemoglobin 7.3 g/dl; and Ca +  + 1.21 mmol/L. As sudden cardiac arrest occurred due to severe respiratory acidosis, we decided to initiate V-V ECMO. Our hospital has an adult ECMO centre, and it is 300 km away from our nearest paediatric ECMO centre, approximately a 3.5-h drive. To prevent death, we decided to use small adult ECMO tubes. ECMO was initiated percutaneously in the left femoral vein, and an incision was made in the right internal jugular vein (MAQUET 2050, Cardiopulmonary GmbH BE-PLS, Germany; left femoral vein: 15 Fr/5 mm single-stage drainage cannula, MAQUET, Germany; right internal jugular vein: 14 Fr/ZX 4.7 return cannula, Changzhou Kangxin Medical Equipment Co., Ltd., China). The blood flow was 1.7 L/min, sweep gas was 1.5 L/min, and FiO_2_ was 100%. Cardiac arrest occurred again after ECMO, and we immediately administered cardiopulmonary resuscitation. Blood gas analysis results at this time were as follows: pH, 6.84; P_a_CO_2_, 72 mmHg; P_a_O_2_, 61 mmHg; Lac, 9.7 mmol/L; K + , 9.8 mmol/L; haemoglobin 7.3 g/dl; and Ca +  + 1.21 mmol/L. Insulin (2 U) were added to the glucose injection (10%, 100 ml), and sodium bicarbonate (5%, 32 ml) and calcium chloride injection (3%, 0.1 g) were administered immediately. Spontaneous sinus rhythm was restored after 18 min. An exploratory thoracotomy was performed successfully; the root of the right middle lobe bronchus was found to be ruptured (Fig. 2), as was a branch of the right middle lobe artery. Right middle lobectomy and right middle bronchoplasty were performed. The patient was admitted to the intensive care unit (ICU) after the operation. Mechanical ventilation and ECMO were continued, and we adjusted the ventilator parameters as follows: FiO_2_ at 30%; positive end expiratory pressure (PEEP) at 10 cmH_2_O; respiratory rate at 12 times/minute, and tidal volume at 6 mL/kg. Oxygen saturation was between 98 and 100%. Along with mild hypothermia for brain protection (34-36 °C for 30 h), piperacillin sodium and tazobactam injection for the prevention of infection, methylprednisolone injection (16 mg q12 h) to reduce pulmonary exudation, and norepinephrine 0.15 µg/kg/min to maintain blood pressure were applied. On postoperative day 2, he became conscious and was responsive. Due to the traumatic wet lung on the left and secondary pulmonary infection, ECMO was withdrawn on the 6th day after the operation, with a total ECMO time of 137 h. Mechanical ventilation was withdrawn on postoperative day 11. On postoperative day 12, chest CT showed a mass of a high-density shadow in the upper lobe of the left lung with cavitation, which was considered a large traumatic pseudocyst (Fig. 3). The patient left the ICU on postoperative day 16 and was discharged from the hospital on postoperative day 31 without neurological deficit. He is able to communicate and play normally. The timeline of the treatment process is shown in Fig. 4.

## Discussion and conclusion

Traumatic bronchial rupture in children is often caused by blunt chest trauma, which is rare making up only 0.2–0.8% of trauma cases reaching the emergency department,but often life-threatening [1–3]. A review of the literature on tracheobronchial rupture [4] concluded that motor vehicle accidents are the most frequent mechanism of injury (59%), that the injury occurs within 2 cm of the carina in 76% of patients, and that 43% occur within the first 2 cm of the right main bronchus.

The cricoid cartilage and tracheal carina are fixed anatomically, with the two lungs on either side; when the chest is compressed forward and backwards, the lungs are pushed sideways, and the two bronchi near the carina produce a shear force, leading to bronchial rupture. When the chest wall is squeezed suddenly, the glottic reflex is closed, the thorax shrinks, and the internal pressure of the bronchus increases sharply. If the pressure exceeds the tolerance limit of the bronchus, the bronchus ruptures. The bronchus walls are weak in children, who often hold their breath at the moment they are injured due to a startle response; this can increase the airway pressure and relative movement of the bronchus and lung, which is a special feature of traumatic bronchus rupture in children.

Children with bronchial rupture should be treated as soon as possible after diagnosis. Early surgery can quickly restore bronchial continuity, terminate pneumothorax, preserve lung tissue as much as possible, and restore lung function. Surgical repair should be performed according to the degree of bronchial rupture. In severe cases, lobectomy is needed. ECMO can be employed to provide pulmonary support after blunt chest trauma. However, severe trauma injuries are considered to be a contraindication for ECMO because of the risk of unstoppable bleeding or intracranial haemorrhage. In adult patients, V-V ECMO can be used for supportive treatment of intrabronchial haemorrhage [5] and auxiliary measures for surgical repair after iatrogenic [6] or traumatic bronchial rupture [7]. Fortenberry and others [8] first proposed that ECMO be used to provide support for posttraumatic respiratory failure or acute respiratory distress syndrome in children.

Most previous articles reported that paediatric patients were treated at specialized paediatric ECMO centres [2, 9, 10]. The success of the present rescue shows that adult ECMO centres can also provide pulmonary support for children in an emergency. Ballouhey and others [11] reported that they successfully rescued several children with traumatic bilateral bronchial rupture by ECMO. They believed that ECMO should be implemented as soon as possible after chest deflation and mechanical ventilation in children with severe bronchial rupture. ECMO can be initiated before surgery, when instability occurs during thoracic surgery or when mechanical ventilation is expected to be difficult. ECMO can also be established after surgical repair to prevent continuous ventilatory difficulties or barotrauma. ECMO was started after cardiac arrest in our patient. We also consider that it may have been possible to avoid the first cardiac arrest if ECMO had been performed when the oxygenation index was first found to be extremely low. During ECMO, the boy again experienced cardiac arrest due to severe hyperkalaemia and acidosis. Four hundred millilitres of red blood cells were prefilled into the ECMO tube; however, 800 millilitres of stock red blood cells were used afterwards, which resulted in hyperkalaemia. Studies have shown that the longer the storage time of red blood cells is, the higher is the concentration of K + and lactic acid [12]. In children, prefilling a large volume of stored red blood cells directly can lead to low pH and high K + and lactic acid concentrations, which increase myocardial irritability, reduce the ventricular fibrillation threshold, and may cause myocardial weakness, hypotension, and ventricular fibrillation. If cardiac arrest occurs immediately after the initiation of ECMO, hyperkalaemia needs to be ruled out, and it is very important to perform blood gas analysis and treat hyperkalaemia in a timely manner. Furthermore, the use of red blood cells cleaned by a blood recovery machine or a fresh red blood cell for prefilling should be considered for paediatric patients. ECMO played a vital role in the successful treatment of bronchial rupture in this patient. However, there are many lessons to be learned in the implementation process. Overall, the application of ECMO in children is different from that in adults and requires more refined management.

In summary, V-V ECMO can be considered for support in children with severe acute respiratory failure after bronchial rupture. In an emergency, V-V ECMO can be carried out effectively in a qualified and experienced adult ECMO centre.

## Data Availability

The datasets used and/or analysed during the current study are available from the corresponding author on request.

## Abbreviations

V-V: Veno-venous
ECMO: Extracorporeal membrane oxygenation
HR: Heart rate
Bpm: Beats per minute
Bp: Blood pressure
RR: Respiratory rate
S_P_O_2_: Oxygen saturation
CT: Computed tomography
P_a_CO_2_: Carbon dioxide partial pressure
P_a_O_2_: Oxygen partial pressure
P_et_CO_2_: End-tidal carbon dioxide partial pressure
Lac: Lactic acid
ICU: Intensive care unit
PEEP: Positive end expiratory pressure
